# Genome-Wide Identification of the *Polygalacturonase* Gene Family and Its Potential Association with Abscission Zone in *Capsicum annuum* L.

**DOI:** 10.3390/genes16050579

**Published:** 2025-05-14

**Authors:** Lei He, Chen Lu, Xi Yan, Sha Yang, Peng Zhou, Wei Lai, Jianwen He

**Affiliations:** 1 Pepper Research Institute, Guizhou Academy of Agricultural Sciences, Guiyang 550025, China; cahelei955@126.com (L.H.);; 2 Guizhou Key Laboratory of Molecular Breeding for Characteristic Horticultural Crops, Guiyang 550025, China; 3 College of Horticulture, Hunan Agricultural University, Changsha 410125, China

**Keywords:** *Capsicum annuum*, *polygalacturonase*, gene family, pedicel abscission susceptibility, qRT-PCR

## Abstract

Background: *Polygalacturonase* (*PG*) genes regulate plant organ abscission by degrading pectin in the cell wall. However, their association with pedicel abscission susceptibility in pepper remains poorly understood. Methods: 47 *CaPG* genes were identified were identified in the ‘Zunla1’ genome and characterized by structural, evolutionary, and comparative genomic analyses. Their expression profiles across various tissues and fruit development stages were examined using transcriptome data. Ethephon treatment and qRT-PCR were employed to assess gene responses during ethylene-induced pedicel abscission. Results: The 47 *CaPG* genes were distributed across 12 chromosomes, with *CaPG1* to *CaPG5* unanchored. Most proteins were hydrophilic, nuclear-localized, and had promoters enriched in light-responsive elements. Collinearity analysis revealed limited segmental duplication, and Ka/Ks values indicated strong purifying selection. Phylogenetic and collinearity analyses showed that *CaPG* genes are more closely related to those in tomato than in *Arabidopsis* or maize. Expression profiling revealed tissue- and stage-specific patterns, with 21 *CaPG* genes associated with pedicel abscission susceptibility. Ethephon treatment enhanced abscission and upregulated several *CaPG* genes. Conclusions: This study offers insights into the *CaPG* gene family’s structure, evolution, and function. Specific *CaPG* genes likely contribute to ethylene-mediated pedicel abscission, providing potential targets for improving fruit-retention traits in pepper.

## 1. Introduction

Pepper (*Capsicum annuum* L.) is a globally important horticultural crop valued for its nutritional, economic, and processing attributes [1,2]. A key agronomic trait is pedicel abscission susceptibility, which affects both harvesting efficiency and post-harvest processing by determining how easily the fruit detaches from the plant and the pedicel from the fruit. Similar traits are studied in other crops, such as sweet cherry, where stem–fruit abscission is crucial for mechanical harvesting and regulated by ethylene, and in bananas, where ripening affects fruit stalk detachment and processing quality [3,4].

Pedicel abscission is influenced by hormonal signals, transcription factors, and environmental conditions. Ethylene promotes abscission by activating gene expression in the abscission zone, often after auxin levels drop, as seen in tomatoes [5]. Jasmonic acid also promotes abscission, while auxin inhibits it. The transcription factor *SlHB15A* regulates this interaction by controlling JA-Ile biosynthesis [6]. Other transcription factors like *PsJOINTLESS* in pea and *LcERF2* in litchi activate genes for cell wall remodeling, aiding abscission [7,8]. Environmental stresses, such as cold in apples and carbohydrate shortage in longan, increase abscission by disrupting hormones and triggering ROS, which enhance cell wall-degrading enzyme expression [9,10].

*Polygalacturonases* (PGs), a class of cell wall-modifying enzymes that degrade pectin, have been primarily associated with fruit softening [11,12,13,14]. However, recent studies have revealed their role in organ abscission. Since abscission requires significant cell wall changes, PGs are seen as crucial regulators [15,16]. In citrus, *PGs* like *CitPG2*, *CitPG29*, and *CitPG34* are prominently expressed during fruit abscission and are influenced by ethylene and auxin [17]. Similarly, in tomatoes, PG activity increases in the abscission zones of flowers and leaves, indicating a conserved function across species [18].

Despite the growing recognition of *PG* genes in organ abscission, their functional roles in pepper pedicel abscission remain largely unexplored. Prior research in *Capsicum* has primarily focused on PG involvement in fruit ripening and softening. PG activity and expression patterns vary across developmental stages in sweet pepper, and a single gene linked to fruit abscission has been identified [19,20,21,22]. To address this knowledge gap, we conducted a comprehensive genome-wide identification and analysis of the *PG* gene family in *Capsicum annuum*. We performed a genome-wide identification and characterization of the *PG* gene family in *Capsicum annuum* and conducted gene expression profiling across various tissues and developmental stages, with particular attention to pedicel tissues exhibiting different levels of abscission sensitivity. Furthermore, we examined the ethylene responsiveness of selected *CaPG* genes using quantitative real-time PCR. This study aims to elucidate the regulatory roles of *PG* genes in pepper pedicel abscission and to identify candidate genes for improving fruit detachment traits, thereby supporting breeding strategies for enhanced mechanical harvesting and post-harvest processing efficiency. We hypothesize that specific *CaPG* genes play key regulatory roles in pedicel abscission and that their expression is modulated by ethylene signaling pathways.

## 2. Results

### 2.1. Identification and Characteristics of CaPGs

A total of 47 *CaPG* genes were identified from the *Capsicum annuum* ‘Zunla 1’ genome. These genes were labeled *CaPG1* to *CaPG47* based on their chromosomal positions. The proteins encoded by these genes range in size from 261 to 598 amino acids. Their molecular weights range from 28,032.14 Da to 63,472.85 Da. The isoelectric points of the proteins span from 4.95 to 9.80. The instability indices vary from 25.29 to 55.72, with 14 proteins classified as unstable. Hydrophilicity analysis revealed that 42 proteins are hydrophilic, while 5 are hydrophobic. Subcellular localization predictions indicate that the *CaPG* proteins are distributed across various cellular compartments, with a predominant localization in the nucleus (Table 1 and Table S1).

### 2.2. Phylogenetic Analysis and Classification of CaPGs

**Figure 1:**
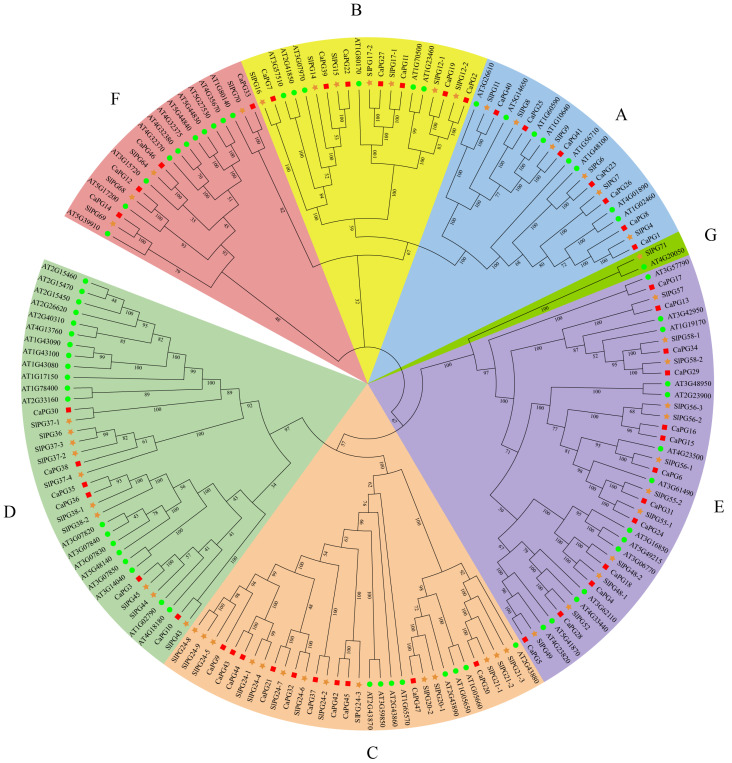
Phylogenetic tree of the *Capsicum annuum*, *Solanum lycopersicum*, and *Arabidopsis thaliana PG* family. A phylogenetic tree was generated based on Neighbor-Joining (NJ) analysis, utilizing 1000 bootstrap replicates for accuracy. According to the tree, all PGs were divided into seven groups (**A**–**G**).

To further elucidate the evolutionary relationships within the PG gene family in *Capsicum annuum*, a comprehensive phylogenetic analysis was conducted. This analysis used 169 PG protein sequences, which were derived from *Capsicum annuum* (47 sequences), *Arabidopsis thaliana* (68 sequences), and *Solanum lycopersicum* (54 sequences). The resulting phylogenetic tree classified these PG proteins into seven distinct subfamilies, labeled A through G. The PG proteins from *C. annuum* were distributed among six subfamilies (A–F), indicating the significant diversification of the PG family across the examined species. Furthermore, the analysis revealed a closer evolutionary affinity between the PG proteins of *C. annuum* and *S. lycopersicum* (Figure 1).

### 2.3. Distribution of CaPG Genes Across Chromosomes

**Figure 2:**
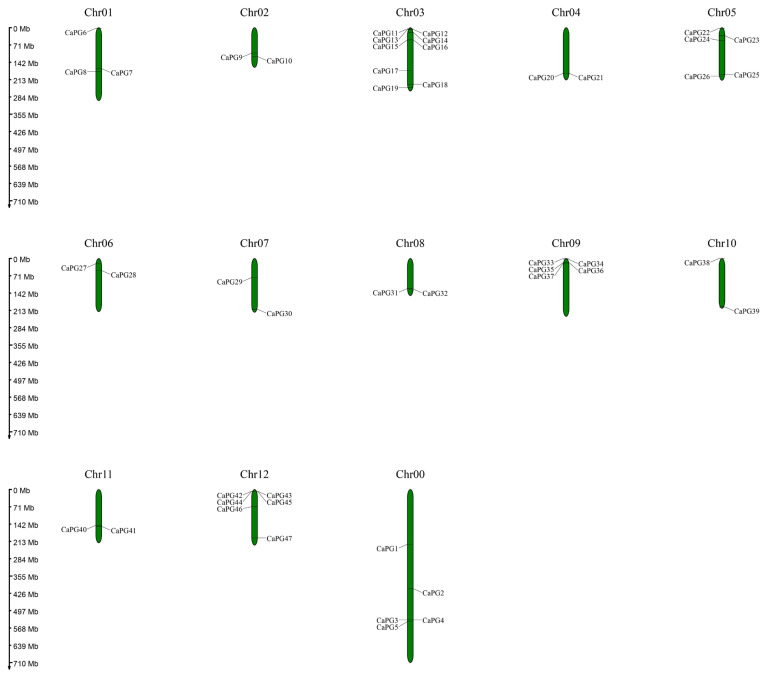
Chromosome location of *CaPG* genes in pepper.

The identified *CaPG* genes were distributed across all chromosomes of the *Capsicum annuum* genome, except for five genes (*CaPG1* to *CaPG5*). These five genes were located on an unassigned chromosome, designated as chromosome 0. Among the 12 annotated chromosomes, chromosome 3 contained the highest number of *CaPG* genes, with a total of nine (*CaPG11* to *CaPG19*). In contrast, chromosomes 2, 4, 6, 7, 8, 10, and 11 each contained only two *CaPG* genes. These chromosomes had the lowest gene counts (Figure 2).

### 2.4. Gene Structure and Conserved Motif Composition

**Figure 3:**
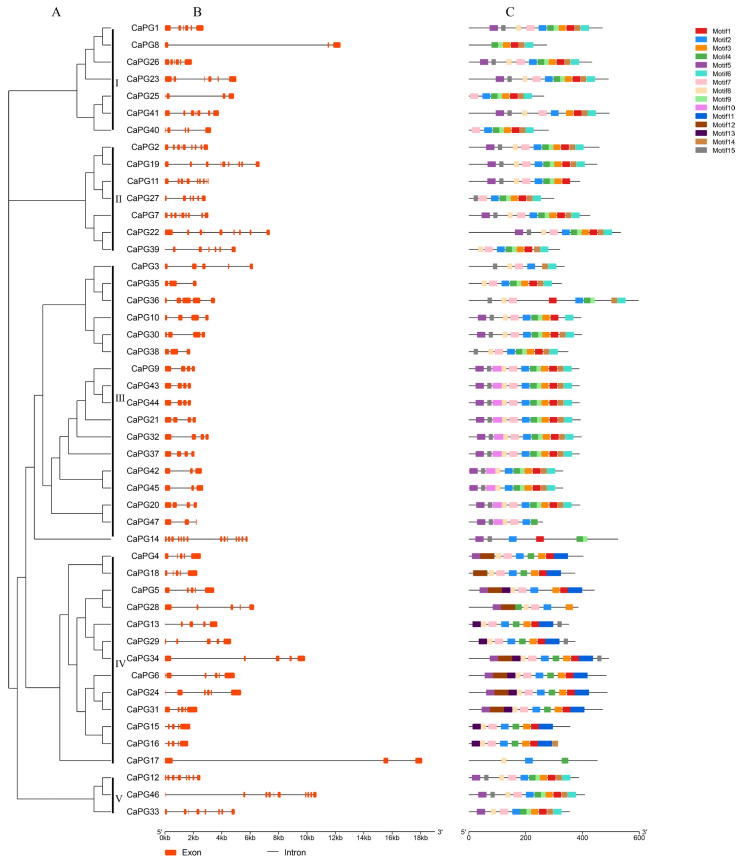
Phylogenetic relationships, gene structures, and conserved protein motifs of *CaPGs*. (**A**) A phylogenetic tree of *CaPGs* was constructed using the maximum likelihood method in MEGA, based on 1000 bootstrap replicates. (**B**) Exon–intron structures of *CaPG* genes are shown, where orange boxes represent exons and black lines represent introns. (**C**) The distribution of conserved motifs within the *CaPG* proteins is illustrated, with each of the 15 motifs indicated by differently colored boxes.

Figure 3 delineates the phylogenetic relationships, gene structures, and conserved motif compositions of the *CaPG* gene family. Panel (A) presents a phylogenetic tree of 47 *CaPG* genes, which are grouped into five distinct clades, indicating evolutionary divergence and potential functional differentiation. Panel (B) depicts the exon–intron structures of the *CaPG* genes, revealing that most genes comprise between 2 and 6 exons; however, certain genes, such as *CaPG8* and *CaPG17*, display extended intronic regions, suggesting possible regulatory complexity or alternative splicing events. Panel (C) highlights the distribution of 15 conserved motifs within the *CaPG* protein sequences, with members of the same clade generally exhibiting similar motif compositions, suggesting conserved functional domains within each subgroup.

### 2.5. Analysis of Cis-Acting Elements

**Figure 4:**
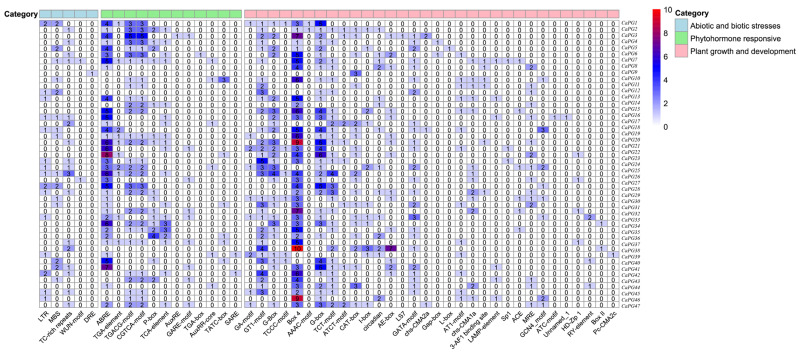
Distribution of cis-acting elements in the *CaPG* gene family. Forty-nine cis-acting element types were grouped into three categories. The grid numbers show element counts, with colors from light blue to red indicating higher quantities.

The analysis of cis-acting elements within the PG gene family identified “plant growth and development” elements as the most prevalent, with light-responsive elements being particularly common, among which Box-4 was the most frequent, followed by G-box. This underscores their significant roles in morphogenesis and organ differentiation. Elements categorized under “phytohormone responsive”, including ABRE, which is associated with abscisic acid (ABA), were the second most common, indicating their potential involvement in the coordinated regulation of hormonal responses. Conversely, elements linked to “abiotic and biotic stress” responses, such as MBS and LTR, were less frequently observed but may still play a role in stress adaptation (Figure 4).

### 2.6. Intraspecific Collinearity Analysis of CaPG Genes

**Figure 5:**
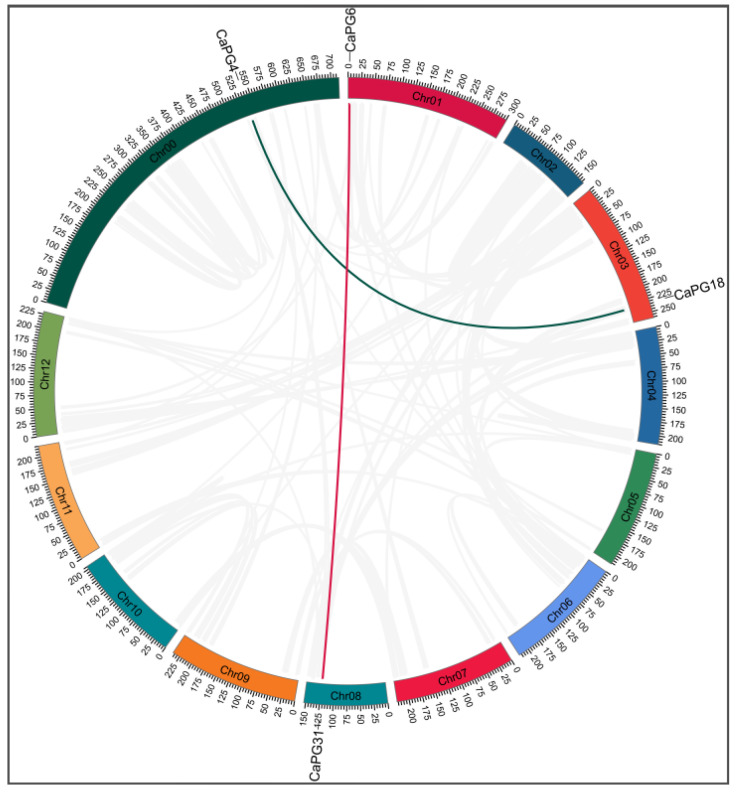
Collinearity analysis of *CaPG* genes in *Capsicum annuum*. Red and green lines represent collinear relationships between *CaPG* gene pairs (*CaPG4*–*CaPG18* and *CaPG6*–*CaPG31*), while gray lines denote genome-wide duplication events.

To elucidate the evolutionary history of the *CaPG* gene family, a collinearity analysis was performed on the *Capsicum annuum* genome (Figure 5). This analysis identified two distinct collinear gene pairs, *CaPG4*–*CaPG18* and *CaPG6*–*CaPG31*. The findings suggest that segmental duplication events have been instrumental in the expansion and diversification of the *CaPG* gene family. Additionally, the presence of extensive gray lines, indicative of genome-wide duplication events, underscores the dynamic and complex evolutionary processes shaping the pepper genome.

The Ka/Ks analysis of the two collinear *CaPG* gene pairs (*CaPG4*–*CaPG18* and *CaPG6*–*CaPG31*) demonstrated that both pairs exhibit Ka/Ks ratios significantly below 1 (0.1578 and 0.1123, respectively), indicating that they have undergone strong purifying selection to preserve functional conservation. Based on their collinear genomic locations, both pairs are classified as products of segmental duplication events (Table 2).

### 2.7. Synteny Analysis Among CaPG Genes

**Figure 6:**
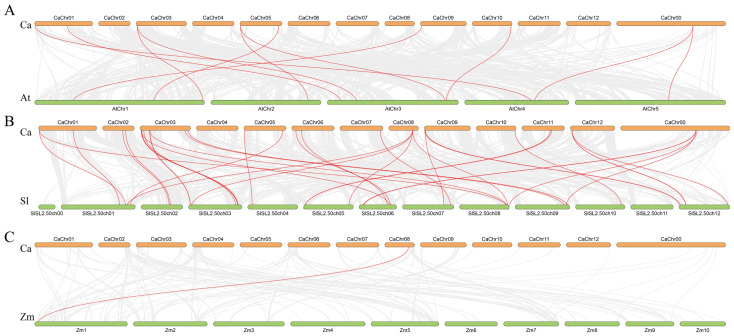
Collinearity analysis plot of *Capsicum annuum* with *Arabidopsis thaliana*, *Solanum lycopersicum*, and *Zea mays*. (**A**) *Capsicum annuum* and *Arabidopsis thaliana*. (**B**) *Capsicum annuum* and *Solanum lycopersicum*. (**C**) *Capsicum annuum* and *Zea mays*. Collinear blocks are marked with gray lines, while collinear gene pairs with PG genes are highlighted with red lines.

To investigate the evolutionary history of the *CaPG* gene family, we performed a comparative synteny analysis between *Capsicum annuum* and three other species: *Arabidopsis thaliana*, *Solanum lycopersicum*, and *Zea mays* (Figure 6). Our analysis identified 11 homologous gene pairs between *CaPG* and *AtPG* genes, 32 pairs between *CaPG* and *SlPG* genes, and only a single pair between *CaPG* and *ZmPG* genes. Significantly, a greater number of collinear relationships were observed with the dicotyledonous species (*A. thaliana* and *S. lycopersicum*) compared to the monocotyledonous species (*Z. mays*). This suggests that the expansion of the *CaPG* gene family likely occurred prior to the divergence of dicots and monocots, indicating a closer evolutionary relationship between *CaPG* genes and those of dicot species.

### 2.8. Expression Profiles of CaPG Genes in Different Tissues and Fruit Developmental Stages

**Figure 7:**
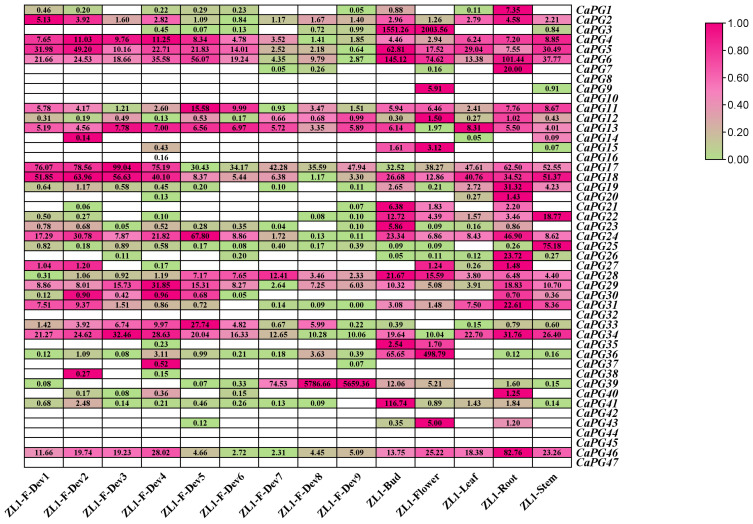
Expression profiles of *CaPG* genes across diverse tissues and fruit development stages in *Capsicum annuum*. RNA-seq data from root, stem, leaf, bud, flower, and fruit tissues were analyzed to determine transcript abundance. Fruit development was divided into nine stages: five early stages (ZL1-Dev1–ZL1-Dev5: 0–1 cm, 1–3 cm, 3–4 cm, 4–5 cm, and mature green), the breaker stage (ZL1-Dev6: color transition), and three post-breaker stages (ZL1-Dev7–ZL1-Dev9: 3, 5, and 7 days after breaker). Expression levels (FPKM) were log_2_-transformed, and a heatmap was generated using TBtools-Ⅱ v2.225. High and low transcript levels are represented by magenta and green, respectively; white indicates no detectable expression.

The expression profiles of 47 *CaPG* genes across various tissues and developmental stages were analyzed, revealing distinct and tissue-specific transcription patterns (Figure 7, Table S2). Several genes exhibited high expression levels in specific tissues. For instance, *CaPG1*, *CaPG7*, *CaPG19*, *CaPG20*, *CaPG26*, *CaPG31*, *CaPG40*, and *CaPG46* were predominantly expressed in the root (ZL1-Root). In contrast, *CaPG3*, *CaPG15*, *CaPG35*, and *CaPG36* showed elevated expression in both the bud (ZL1-Bud) and flower (ZL1-Flower), with *CaPG21*, *CaPG23*, and *CaPG41* being specifically upregulated in the bud. Genes such as *CaPG2* and *CaPG4*–*CaPG6* were highly expressed during the early stages of fruit development (ZL1-F-Dev1 to ZL1-F-Dev7), while *CaPG7* also had significant expression in the flower (ZL1-Flower). *CaPG27* was notably upregulated in both the root and flower, whereas *CaPG25* was predominantly expressed in the stem (ZL1-Stem). Additionally, *CaPG17* and *CaPG18* showed strong expression across multiple tissues and early fruit development stages (ZL1-F-Dev1 to ZL1-F-Dev4). *CaPG22* exhibited consistent high expression across all tissues, while *CaPG11*–*CaPG13*, *CaPG28*–*CaPG29*, and *CaPG34* displayed variable expression across tissues and fruit developmental stages. *CaPG33* showed a progressive increase in expression from ZL1-F-Dev1 to ZL1-F-Dev5. In contrast, genes such as *CaPG8*, *CaPG10*, *CaPG14*, *CaPG16*, *CaPG32*, *CaPG37*, *CaPG38*, *CaPG42*, *CaPG44*, *CaPG45*, and *CaPG47* exhibited no detectable expression in any of the tissues or developmental stages. Notably, *CaPG39* was sharply upregulated in the later stages of fruit development, with a dramatic increase in expression from ZL1-F-Dev8 to ZL1-F-Dev9. These findings suggest that the *CaPG* gene family is involved in diverse tissue-specific and developmental stage-specific functions, highlighting the complexity of their roles in pepper plant development.

### 2.9. Expression Patterns of CaPG Genes Related to Pedicel Abscission Susceptibility

**Figure 8:**
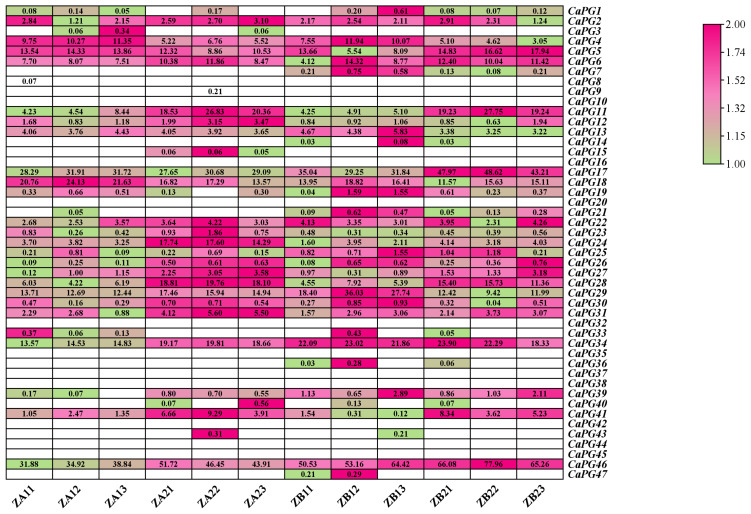
Heatmap of *CaPG* genes expression in tissues with contrasting susceptibility to pedicel abscission. RNA-seq data were used to examine *CaPG* transcript levels in samples from high-susceptibility zones (ZA11–ZA13, ZB21–ZB23) versus low-susceptibility zones (ZB11–ZB13, ZA21–ZA23). Expression values (FPKM) were log_2_-transformed, and the heatmap was generated with TBtools-Ⅱ. Magenta denotes high expression; green, low expression; and white, no detectable expression.

Analysis of the heatmap in Figure 8 revealed that 21 *CaPG* genes were expressed in tissues with differing susceptibility to pedicel abscission (Figure 8, Table S2). Among them, *CaPG2*, *CaPG4*, *CaPG5*, *CaPG6*, *CaPG11*, *CaPG12*, *CaPG13*, *CaPG17*, *CaPG18*, *CaPG23*, *CaPG24*, *CaPG25*, *CaPG27*, *CaPG28*, *CaPG31*, *CaPG34*, *CaPG41*, and *CaPG46* showed differential expression between the high-susceptibility zones (ZA11–ZA13 and ZB21–ZB23) and the low-susceptibility zones (ZA21–ZA23 and ZB11–ZB13). In particular, *CaPG22*, *CaPG29*, and *CaPG39* exhibited markedly higher expression levels in the low-susceptibility zones (ZA21–ZA23 and ZB11–ZB13) compared with the high-susceptibility zones (ZA11–ZA13 and ZB21–ZB23), suggesting a potential role in maintaining pedicel integrity and preventing abscission.

### 2.10. qRT-PCR Validation of CaPG Genes Involvement in Ethylene-Induced Pedicel Abscission

**Figure 9:**
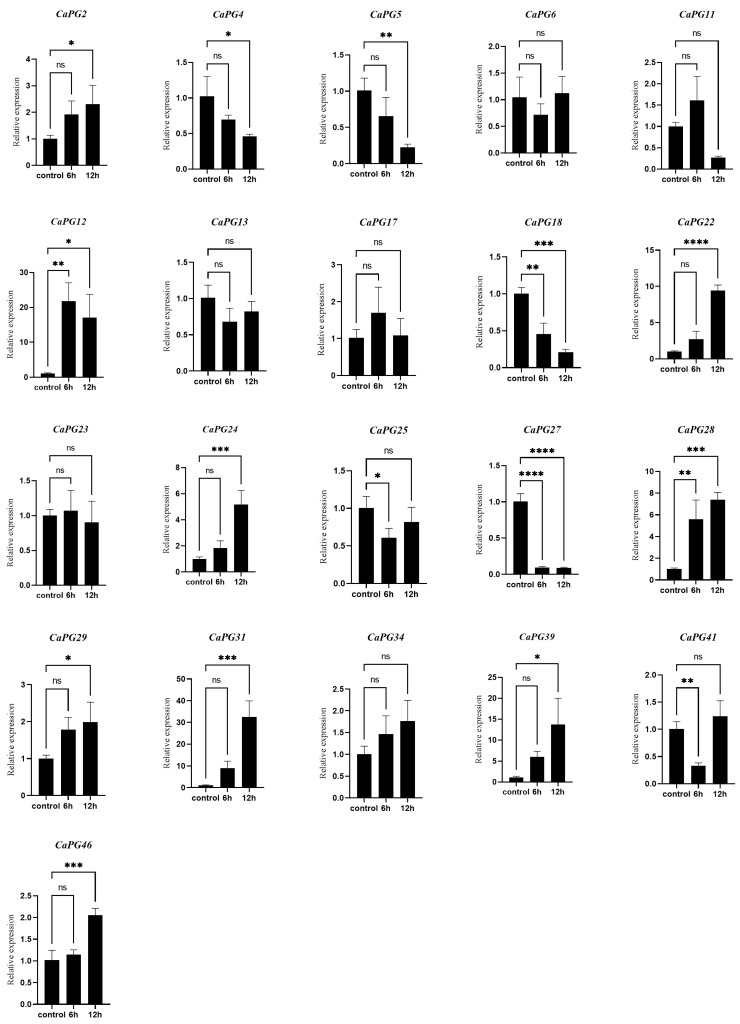
qRT-PCR analysis of 21 *CaPG* genes in the pedicel abscission zone following ethephon treatment. Transcript levels were measured at 0 h (control), 6 h, and 12 h after ethephon application. Data are presented as means ± SD of three biological replicates. Statistical significance compared with 0 h: * *p* ≤ 0.05; ** *p* ≤ 0.005; *** *p* ≤ 0.0005; **** *p* ≤ 0.0001; ns, not significant.

qRT-PCR analysis was performed to validate the expression patterns of 21 *CaPG* genes in response to ethylene treatment (Figure 9). The results showed that the transcript levels of *CaPG2*, *CaPG22*, *CaPG24*, *CaPG29*, *CaPG31*, *CaPG34*, *CaPG39*, and *CaPG46* increased at 6 and 12 h after ethephon treatment. *CaPG12* exhibited a significant upregulation at both 6 h and 12 h post-treatment. In contrast, *CaPG11*, *CaPG17*, and *CaPG23* displayed a transient increase at 6 h followed by a decrease at 12 h. Similarly, *CaPG6*, *CaPG13*, *CaPG25*, and *CaPG41* showed an initial decrease at 6 h, followed by an increase at 12 h. Meanwhile, *CaPG4* and *CaPG5* exhibited reduced expression at 6 h, with a significant further reduction at 12 h. In addition, *CaPG18* and *CaPG27* showed a marked downregulation at both 6 h and 12 h after ethephon treatment. These results suggest that multiple *CaPG* genes are dynamically regulated by ethylene and may play diverse roles during pedicel abscission.

## 3. Discussion

PGs are integral to cell wall remodeling and are intricately linked to organ abscission, fruit softening, and pathogen responses in plants [23,24,25,26]. This study presents a comprehensive genome-wide analysis of the *CaPG* gene family in *Capsicum annuum*, offering significant insights into their evolutionary characteristics, expression patterns, and potential regulatory roles in abscission zones and ethylene-induced cell separation processes. A total of 47 *CaPG* genes were identified within the pepper genome, which is fewer than the 68 *PGs* in *Arabidopsis* and 54 in tomato, indicating a relatively conserved expansion pattern among dicots [26,27]. Variation in molecular weights, isoelectric points, and hydrophobicity values among *CaPG* proteins highlights their structural diversity, potentially reflecting functional *specialization* across developmental and stress-responsive pathways [28,29]. Most *CaPG* proteins were predicted to be hydrophilic and localized primarily in the nucleus (Table 1). Phylogenetic analysis categorized *CaPGs* into six subfamilies (A–F), similar to classifications observed in *Arabidopsis* and tomato [26,27], reflecting both functional conservation and lineage-specific divergence within the *Solanaceae* family [30,31]. The chromosomal distribution of *CaPG* genes was uneven, with the largest cluster found on chromosome 3 (Figure 2), a pattern also observed in strawberry, suggesting a conserved genomic arrangement of *PG* genes across some dicots [32].

Gene structure and motif composition analyses revealed conserved consistent exon-intron organization and motif patterns among *CaPG* genes within the same clades (Figure 3). This structural conservation implies functional coherence, while the presence of long introns in genes like *CaPG8* and *CaPG17* may suggest regulatory complexity through alternative splicing or transcriptional modulation [33,34,35,36].

Cis-element analysis uncovered diverse hormone- and stress-responsive elements, suggesting that *CaPG* genes participate in a broad range of biological processes (Figure 4). These findings align with previous evidence showing *PG’s* involvement in fruit ripening, abscission, and stress responses [11,12,13,14,15,16,17,18].

Synteny analysis showed stronger collinearity between pepper and dicots such as *Arabidopsis* and tomato compared to monocots like maize (Figure 6). This supports the hypothesis that *PG* gene family expansion occurred before the monocot–dicot split [26,27,30] and confirms an early expansion event retained in dicot lineages.

Expression profiling demonstrated that *CaPGs* exhibit distinct tissue- and stage-specific expression patterns (Figure 7). For example, *CaPG1*, *CaPG7*, and *CaPG26* exhibit high transcript abundance in roots, whereas *CaPG35* and *CaPG36* show strong expression in floral tissues. This pattern of tissue-specific expression of PGs has been associated with cell wall remodeling during growth and differentiation in other plant species [7,31,32]. Among the *CaPGs* involved in reproductive development, *CaPG2*, *CaPG4*, and *CaPG6* exhibit peak expression during the early stages of fruit development (ZL1-F-Dev1 to ZL1-F-Dev7), which is consistent with their putative roles in pectin degradation and cell wall loosening, key events during fruit expansion and softening [30,31,32]. In contrast, *CaPG39* was significantly upregulated at later stages (ZL1-F-Dev8 to ZL1-F-Dev9), suggesting an involvement in maturation-related cell wall changes and the potential activation of abscission zone cells, paralleling the role of *SlPGs* during tomato ripening [14]. In addition, heatmap analysis of *CaPG* gene expression across tissues with varying susceptibility to pedicel abscission (Figure 8, Table S2) revealed that 21 *CaPG* genes were differentially expressed among these tissues. Notably, *CaPG* genes with higher expression in easy-abscission pedicel tissues, such as *CaPG22*, *CaPG29*, and *CaPG39*, are likely involved in promoting cell separation at the abscission zone, whereas genes with low expression in these tissues may function to delay or suppress abscission. This spatial expression divergence indicates that *CaPGs* participate in the fine-tuning of pedicel abscission zone formation and activation.

Ethylene is known to regulate organ abscission [37,38]. Our qRT-PCR results showed that *CaPG22*, *CaPG29*, and *CaPG39* are ethylene-responsive, with transcript levels significantly increasing after ethephon treatment. These genes may mediate ethylene-induced cell separation in the pedicel abscission zone. Particularly, *CaPG39* (*Capana10g002229*) exhibited both strong ethylene responsiveness and late-stage upregulation, reinforcing its dual role in fruit maturation and abscission. This is in agreement with previous findings identifying *CaPG39* as a candidate gene involved in fruit drop regulation in mature pepper fruit [22]. Combined with its high expression in tissues prone to abscission, *CaPG39* may act as a key mediator in ethylene signaling cascades that activate cell wall degradation within the abscission zone. The distinct expression profiles of *CaPGs* across tissues with varying abscission susceptibility highlight their functional diversification in modulating the timing and strength of abscission. The findings indicate that modulating the expression of specific *CaPG* genes, particularly those associated with ethylene-induced cell separation, may serve as a genetic approach to delay or control pedicel abscission in peppers. To validate these findings, future research should prioritize functional analyses of candidate genes, including *CaPG22*, *CaPG29*, and *CaPG39*, employing methodologies such as gene knockout, overexpression, or CRISPR/Cas9-mediated editing. Understanding the functional roles of these genes is essential for mitigating preharvest fruit drop and enhancing the efficiency of mechanized harvesting, thereby providing significant benefits for breeding programs aimed at developing varieties that are easier to harvest.

## 4. Materials and Methods

### 4.1. Plant Growth and Treatments

*Capsicum annuum* cv. Zunla-1 plants were cultivated under field conditions at the Zunyi Experimental Station (N 27°44′, E 107°12′) of the Pepper Research Institute, Guizhou Academy of Agricultural Sciences. At the green mature fruit stage, healthy and uniformly growing plants were selected for ethylene treatment. To assess the ethylene responsiveness of *CaPG* genes, a 40% ethephon solution was diluted 100-fold with distilled water and uniformly applied to the aerial parts of the plants until runoff was achieved. Sampling was conducted at three time points: immediately before ethephon application (0 h, control), and at 6 h and 12 h post-treatment. At each time point, 30 plants were treated, and tissues from the fruit pedicel abscission zone were collected from 10 plants per biological replicate. Fruits from each group of 10 plants were pooled to form one replicate, yielding three independent biological replicates per time point. All samples were flash-frozen in liquid nitrogen and stored at −80 °C for RNA extraction.

### 4.2. Identification and Chromosomal Distribution of CaPG Genes

The genome assembly, protein sequences, and corresponding annotation files of *Capsicum annuum* (Zunla-1_v2.0) were retrieved from the Sol Genomics Network database *(*https://solgenomics.net/ftp/genomes/Capsicum_annuum/C.annuum_zunla/, accessed on 13 August 2024) [39]. To identify *PG* gene family members in *C. annuum*, a set of 122 previously characterized PG protein sequences—68 from *A. thaliana* and 54 from *S. lycopersicum*—were compiled as reference sequences. These reference sequences were used to construct a Hidden Markov Model (HMM) profile using HMMER v3.0. Following this, the HMM profile was employed to query the *C. annuum* proteome for potential *PG* gene family members [40]. To ensure a comprehensive identification, a complementary BLASTP alignment (NCBI-BLAST v2.10.1+) was also performed against the *C. annuum* protein database using the same reference PG sequences, with an E-value threshold set to 1 × 10^−5^. The results obtained from both the HMM and BLASTP approaches were subsequently merged to produce a unified set of candidate PG proteins. To verify the identity of these candidates, all sequences were subjected to domain architecture analysis using PfamScan (v1.6) in combination with the Pfam-A database (v33.1). Only those sequences that harbored the characteristic glycoside hydrolase family 28 domain (PF00295) were retained as bona fide members of the *CaPG* gene family [41]. Finally, the chromosomal positions of the identified *CaPG* genes were extracted from the genome annotation files and visualized using the MG2C online tool (http://mg2c.iask.in/mg2c_v2.1/, accessed on 13 August 2024) [42].

### 4.3. Physicochemical Properties of CaPG Proteins

The physicochemical characteristics of the predicted *CaPG* protein sequences were analyzed using the ProtParam tool available on the ExPASy server (http://web.expasy.org/protparam/, accessed on 15 August 2024) [43]. For each protein, parameters including amino acid length, molecular weight (MW), theoretical isoelectric point (pI), grand average of hydropathicity (GRAVY), aliphatic index, and instability index were calculated.

### 4.4. Subcellular Localization Prediction and Signal Peptide Analysis of CaPG Proteins

The subcellular localization of the predicted *CaPG* proteins was analyzed using the ProtComp tool available on the Softberry server (http://www.softberry.com/berry.phtml?topic=protcomp&group=help&subgroup=proloc, accessed on 18 August 2024) [44].

### 4.5. Analysis of Conserved Motifs and Gene Structures

The conserved motifs of *CaPG* protein sequences were identified using the MEME Suite (version 5.0.5; http://meme-suite.org/, accessed on 19 August 2024), a widely used tool for discovering statistically significant sequence motifs [45]. The analysis was conducted using the following parameters: mode set to ZOOPS (zero or one occurrence per sequence), the number of motifs limited to 15 (-nmotifs 15), and the motif width constrained between 6 and 50 amino acids (-minw 6 - maxw 50). The input sequences were analyzed in protein mode (-protein), and the output directory was designated as rea1. The resulting motifs were annotated and visualized to explore the conserved functional domains within the *CaPG* gene family. For gene structure analysis, the exon–intron organization of *CaPG* genes was examined by aligning coding sequences (CDS) with their corresponding genomic DNA sequences. The Gene Structure Display Server (GSDS; http://gsds.cbi.pku.edu.cn/, accessed on 19 August 2024) was used to generate visual representations of the gene structures, enabling the comparison of intron–exon arrangements among different family members [46].

### 4.6. Analysis of Promoters and Phylogeny of CaPG Genes

We extracted the sequence 2000 bp upstream of the transcription start site of the candidate *PG* family gene in pepper using TBtools v2.225 software [47]. These promoter sequences were then submitted to the PlantCARE database (http://bioinformatics.psb.ugent.be/webtools/plantcare/html/, accessed on 20 August 2024) to identify putative cis-acting regulatory elements, including those responsive to hormones, stresses, and developmental cues. For phylogenetic analysis, full-length amino acid sequences of the *CaPG* proteins were aligned using MAFFT software version 7.427 with default parameters [48]. Based on the resulting alignment, a phylogenetic tree was constructed using the Neighbor-Joining (NJ) method implemented in MEGA software (version 10). The parameters were set as follows: substitution model = p-distance, method for handling missing data = Partial Deletion, site coverage cutoff = 50%. Bootstrap analysis was performed with 1000 replicates to assess the reliability of the inferred tree topology, and bootstrap values were used to evaluate the confidence of each branch [49]. The resulting tree was further visualized and annotated using iTOL v6 (https://itol.embl.de/, accessed on 20 August 2024) to better illustrate subgroup classification and evolutionary relationships.

### 4.7. Collinearity Analysis and Visualization of CaPG Genes

Collinearity analysis was conducted to examine the synteny of *CaPG* genes both within *Capsicum annuum* and between *Capsicum annuum* and other species. The species included in the analysis were *Arabidopsis thaliana* (TAIR10 genome release, accessible at http://arabidopsis.org/, accessed on 21 August 2024), *Solanum lycopersicum* (ITAG2.4 genome release from the Sol Genomics Network), and *Zea mays* (B73 RefGen_v4, accessible at http://ensembl.gramene.org/Zea_mays/Info/Index, accessed on 21 August 2024). For within-species synteny analysis, the positions of *CaPG* genes on the chromosomes of *C. annuum* were determined, and the collinearity relationships were visualized using the MCScanX tool [50]. This tool was employed to detect and visualize syntenic blocks and gene duplications within the *C. annuum* genome, providing insights into the evolutionary conservation of PG genes within the species. To examine interspecies synteny, the corresponding *CaPG* gene sequences were aligned with the genomes of *A. thaliana*, *S. lycopersicum*, and *Z. mays* using the same tool. Visualization was performed using TBtools-Ⅱ v2.225 [47].

### 4.8. Tissue-Specific Expression Profiles of CaPG Genes

To investigate the expression patterns of *CaPG* genes in various tissues and during different developmental stages, publicly available transcriptome datasets were utilized. RNA-seq data covering a range of tissues and developmental stages of *Capsicum annuum* (cv. Zunla-1) were retrieved from the NCBI Gene Expression Omnibus (GEO) under accession number GSE45037. Additionally, transcriptome data related to fruit pedicel abscission were obtained from the NCBI Sequence Read Archive (SRA) under the project ID PRJNA1233832.

### 4.9. RT-qPCR

Total RNA was extracted from fruit pedicel abscission zone tissues using the RNAprep Pure Plant Kit (Tiangen Biotech, Beijing, China), according to the manufacturer’s instructions. The quality and concentration of RNA were assessed using a NanoDrop 2000 spectrophotometer (Thermo Fisher Scientific, Waltham, MA, USA), and RNA integrity was verified through 1.0% agarose gel electrophoresis. First-strand cDNA was synthesized from 1 μg of total RNA using the PrimeScript™ RT Reagent Kit with gDNA Eraser (TaKaRa, Dalian, China), following the manufacturer’s protocol. The synthesized cDNA was then diluted 10-fold with nuclease-free water and used as a template for RT-qPCR. Quantitative real-time PCR was conducted using TB Green^®^ Premix Ex Taq™ II (TaKaRa, Dalian, China) on a CFX96 Real-Time PCR Detection System (Bio-Rad, Hercules, CA, USA). The 20 μL reaction system contained 10 μL TB Green Premix, 0.4 μL of each forward and reverse primer (10 μM), 2 μL of diluted cDNA, and 7.2 μL nuclease-free water. The thermal cycling conditions were as follows: 95 °C for 30 s, followed by 40 cycles of 95 °C for 5 s and 60 °C for 30 s. A melting curve analysis was performed at the end of the amplification to confirm product specificity. Gene-specific primers were designed using Primer-BLAST (https://www.ncbi.nlm.nih.gov/tools/primer-blast/, accessed on 25 August 2024) and are listed in Supplementary Table S3. The *EIF5A2* gene of *Capsicum annuum* was used as the internal reference. Relative expression levels were calculated using the 2^−ΔΔCt^ method [51]. Each reaction was performed with three biological replicates and three technical replicates to ensure accuracy and reproducibility.

## 5. Conclusions

This study identified 47 *CaPG* genes in *Capsicum annuum* ′Zunla1′ and examined their potential roles in pedicel abscission. Phylogenetic and structural analyses revealed that *CaPG* genes are evolutionarily conserved and closely related to homologs in *Solanum lycopersicum* and *Arabidopsis thaliana*. The expression profiling revealed tissue- and development-specific expression patterns, and 21 *CaPG* genes were differentially expressed in pedicel zones with varying abscission sensitivities. Following exogenous ethylene treatment, which induced pedicel abscission, qRT-PCR analysis confirmed that several *CaPG* genes showed altered expression, suggesting their potential involvement in the ethylene-mediated abscission process. The study identified the *PG* gene family across the genome, analyzed their expression patterns, and evaluated their ethylene responsiveness in pedicel abscission. It supports the idea that certain *CaPG* genes are regulated by ethylene and influence pedicel abscission. These findings clarify the role of *PG* genes in pepper abscission and lay the groundwork for future studies, such as gene editing, to explore the functions of specific *CaPG* genes. Overall, the results improve our understanding of *PG* gene function and provide insights for breeding better *Capsicum* cultivars.

## Figures and Tables

**Table 1:** List of the 47 *CaPG* genes identified in this study.

Gene Name	Gene ID	Amino Acid Number	Molecular Weight (Da)	Theoretical pI	Instability Index	Aliphatic Index	Average of Hydropathicity (GRAVY)	Subcellular Localization
*CaPG1*	*Capana00g000318*	471	51,283.65	5.66	55.72	78.43	−0.179	chloroplast
*CaPG2*	*Capana00g001601*	460	49,752.13	5.43	32.55	85.17	−0.162	cytoplasm
*CaPG3*	*Capana00g003267*	337	35,659.78	6.52	39.79	89.38	−0.059	extracellular matrix
*CaPG4*	*Capana00g003270*	403	43,430.89	5.54	38.86	85.78	0.049	cytoplasm
*CaPG5*	*Capana00g003407*	443	48,502.59	8.92	34.18	90.61	−0.095	tonoplast
*CaPG6*	*Capana01g000186*	484	53,203.5	5.5	38.59	88.82	−0.095	chloroplast
*CaPG7*	*Capana01g002618*	427	47,497.98	9.43	26.25	85.36	−0.353	chloroplast
*CaPG8*	*Capana01g002826*	275	29,680.37	5.2	46.98	82.22	−0.167	cytoplasm
*CaPG9*	*Capana02g000966*	389	41,790.44	8.61	34.05	86.68	−0.125	nucleus
*CaPG10*	*Capana02g001324*	396	42,839.4	7.18	35.38	85.13	−0.067	extracellular matrix
*CaPG11*	*Capana03g000428*	392	43,288.71	8.82	42.73	85.48	0.026	chloroplast
*CaPG12*	*Capana03g000435*	388	41,598.44	8.59	38.97	90.67	−0.059	nucleus
*CaPG13*	*Capana03g000564*	353	39,730.85	6.66	41.45	87.99	−0.399	cytoskeleton
*CaPG14*	*Capana03g001086*	525	57,077.33	7.8	38.98	93.33	0.022	cytoplasm
*CaPG15*	*Capana03g002144*	357	38,892.43	5.35	36.79	88.99	−0.045	cytoplasm
*CaPG16*	*Capana03g002171*	313	34,204.04	5.35	36.4	89.07	−0.02	cytoplasm
*CaPG17*	*Capana03g003120*	453	50,599.63	8.83	48.51	86.47	−0.201	cytoplasm
*CaPG18*	*Capana03g003665*	374	40,615.51	5.39	39.73	87.51	−0.079	chloroplast
*CaPG19*	*Capana03g004218*	452	49,398.24	4.97	38.09	78.45	−0.312	extracellular matrix
*CaPG20*	*Capana04g002230*	392	42,882.99	9.8	31.47	74.82	−0.215	nucleus
*CaPG21*	*Capana04g002231*	394	42,274.84	8.71	34.46	80.66	−0.124	nucleus
*CaPG22*	*Capana05g000108*	535	59,457.05	5.62	25.29	73.57	−0.741	nucleus
*CaPG23*	*Capana05g000884*	492	53,276.09	4.96	53.51	77.83	−0.211	nucleus
*CaPG24*	*Capana05g001033*	488	53,375.7	5.41	37.8	90.7	−0.096	endoplasmic reticulum
*CaPG25*	*Capana05g002022*	265	28,432.09	8.19	47	73.51	−0.217	chloroplast
*CaPG26*	*Capana05g002171*	434	46,785.2	6.69	51.14	75.74	−0.149	nucleus
*CaPG27*	*Capana06g001141*	300	33,023.9	9.28	40.01	84.1	−0.065	nucleus
*CaPG28*	*Capana06g001746*	386	42,390.74	9.08	41.27	98.42	0.003	cytoplasm
*CaPG29*	*Capana07g000744*	375	42,090.84	7.23	36.51	86.69	−0.332	cytoplasm
*CaPG30*	*Capana07g001965*	399	43,410.72	9.03	36.37	83.01	−0.033	nucleus
*CaPG31*	*Capana08g000965*	472	51,887.04	8.26	38.85	89.64	−0.187	tonoplast
*CaPG32*	*Capana08g001207*	397	42,209.5	5.52	42.32	88.14	−0.038	extracellular matrix
*CaPG33*	*Capana09g000012*	355	38,489.07	5.45	33.72	82.08	−0.155	extracellular matrix
*CaPG34*	*Capana09g000145*	493	55,367.46	8.9	40.58	92.45	−0.145	chloroplast
*CaPG35*	*Capana09g000459*	327	34,860.55	7.47	29.79	80.67	−0.296	nucleus
*CaPG36*	*Capana09g000460*	598	63,472.85	6.69	30.57	83.04	−0.177	nucleus
*CaPG37*	*Capana09g000516*	390	41,929.81	8.87	29.01	79.72	−0.12	nucleus
*CaPG38*	*Capana10g000004*	350	37,695.18	4.95	45.59	83.6	−0.163	nucleus
*CaPG39*	*Capana10g002229*	321	35,227.22	5.69	27.29	73.77	−0.355	nucleus
*CaPG40*	*Capana11g001239*	281	30,660.88	8.41	37.33	86.62	−0.19	nucleus
*CaPG41*	*Capana11g001285*	495	54,293.94	8.97	39.4	75.96	−0.245	chloroplast
*CaPG42*	*Capana12g000384*	332	35,479.08	7.49	26.59	81.54	−0.152	nucleus
*CaPG43*	*Capana12g000385*	390	41,382.38	6.74	37	78.74	−0.105	nucleus
*CaPG44*	*Capana12g000386*	390	41,441.45	7.07	36.02	78.74	−0.113	nucleus
*CaPG45*	*Capana12g000387*	332	35,479.08	7.49	26.59	81.54	−0.152	nucleus
*CaPG46*	*Capana12g001281*	409	44,343.14	5.98	40.42	87.43	−0.048	nucleus
*CaPG47*	*Capana12g002138*	261	28,032.14	8.8	27.91	96.32	0.207	extracellular matrix

**Table 2:** Evolutionary analysis of *CaPG* gene pairs.

Duplicated Pairs	Ka ^1^	Ks ^2^	Ka/Ks	Purifying Selection	Duplicate Type
*CaPG4–CaPG18*	0.1123	0.7115	0.1578	Yes	Segmental
*CaPG6–CaPG31*	0.2493	2.2193	0.1123	Yes	Segmental

^1^ Nonsynonymous substitution rate. ^2^ Synonymous substitution rate.

## Data Availability

The raw RNA-seq data related to fruit pedicel abscission in this study have been deposited in the NCBI Sequence Read Archive under the ID number PRJNA1233832.

## Supplementary Materials

The following supporting information can be downloaded at https://www.mdpi.com/article/10.3390/genes16050579/s1, Table S1: List of all PGs; Table S2: The FPKM from RNA-Seq data of *CaPG* gene family; Table S3: List of primers used in this study.
